# Trilobatin ameliorates dextran sulfate sodium-induced ulcerative colitis in mice via the NF-κB pathway and alterations in gut microbiota

**DOI:** 10.1371/journal.pone.0305926

**Published:** 2024-06-24

**Authors:** Nanbo Wang, Zhaohui Li, Lingling Cao, Zhihua Cui

**Affiliations:** 1 Department of Gastric and Colorectal Surgery, General Surgery Center, The First Hospital of Jilin University, Changchun, China; 2 Changchun People’s Hospital of Jilin Province, Changchun, China; 3 School of Clinical Medical, Changchun University of Chinese Medicine, Changchun, China; 4 The First Hospital of Jilin University, Changchun, China; Kansai Medical University: Kansai Ika Daigaku, Institute of Biomedical Science, JAPAN

## Abstract

**Objective:**

This study aimed to evaluate the effects of trilobatin (TLB) on dextran sulfate sodium (DSS)-induced ulcerative colitis (UC) in mice and further explore the underlying mechanisms from the perspectives of signaling pathway and gut microbiota.

**Methods:**

A mouse model of UC was established using DSS. Trilobatin was administered via oral gavage. Disease severity was assessed based on body weight, disease activity index (DAI), colon length, histological detection, inflammation markers, and colonic mucosal barrier damage. Alternations in the NF-κB and PI3K/Akt pathways were detected by marker proteins. High-throughput 16S rRNA sequencing was performed to investigate the gut microbiota of mice.

**Results:**

In the DSS-induced UC mice, TLB (30 μg/g) treatment significantly increased the body weight, reduced the DAI score, alleviated colon length shortening, improved histopathological changes in colon tissue, inhibited the secretion and expression of inflammation factors (TNF-α, IL-1β, and IL-6), and increased the expression of tight-junction proteins (ZO-1 and occludin). Furthermore, TLB (30 μg/g) treatment significantly suppressed the activation of NF-κB pathway and altered the composition and diversity of the gut microbiota, as observed in the variations of the relative abundances of Proteobacteria, Actinobacteriota, and Bacteroidota, in UC mice.

**Conclusion:**

TLB effectively alleviates DSS-induced UC in mice. Regulation of the NF-κB pathway and gut microbiota contributes to TLB-mediated therapeutic effects. Our study not only identified a novel drug candidate for the treatment of UC, but also enhanced our understanding of the biological functions of TLB.

## Introduction

Ulcerative colitis (UC) is a complex and chronic relapsing inflammatory disease of the colorectal mucosa [1–3]. Typical clinical symptoms of UC include abdominal pain, diarrhea, and bloody stools. Currently, corticosteroids and 5-aminosalicylic acid are used as the first-line drugs for the treatment of UC, and **s**urgical treatment may be inevitable when the outcomes of medical treatments are unsatisfactory. Despite years of research on UC treatment, current clinical therapies for this disease remain insufficient [4]. Owing to the lack of a satisfactory therapeutic regimen, patients with UC frequently require lifelong treatment [5,6]. Previous studies identified UC as a risk factor for colon cancer [7–9]. Therefore, there is an urgent need to identify and develop new and effective agents for the treatment of UC.

Various signaling pathways play key roles in the occurrence and development of many diseases. Research on the related pathways could uncover the regulatory mechanisms underlying these diseases. Studies have shown that activation of both NF-κB and PI3K/Akt pathways is closely associated with the onset and progression of UC [10,11]. In particular, the phosphorylation of p65 and IKB-α leads to the activation of NF-κB pathway and regulation of downstream targets, while phosphorylation of Akt activates the PI3K/Akt pathway [12,13]. Furthermore, growing evidence shows that the composition and distribution of the gut microbiota, which plays an important role in the mucosal immune system, are closely associated with the pathogenesis of UC [14–16]. Moreover, high-throughput sequencing techniques facilitate comprehensive analysis of the structure of the gut microbiota in various animal models, including rats and mice. A previous study highlighted the association between metabolic pathways in UC and the disease-specific composition and distribution of gut microbiota [17].

**Fig 1:**
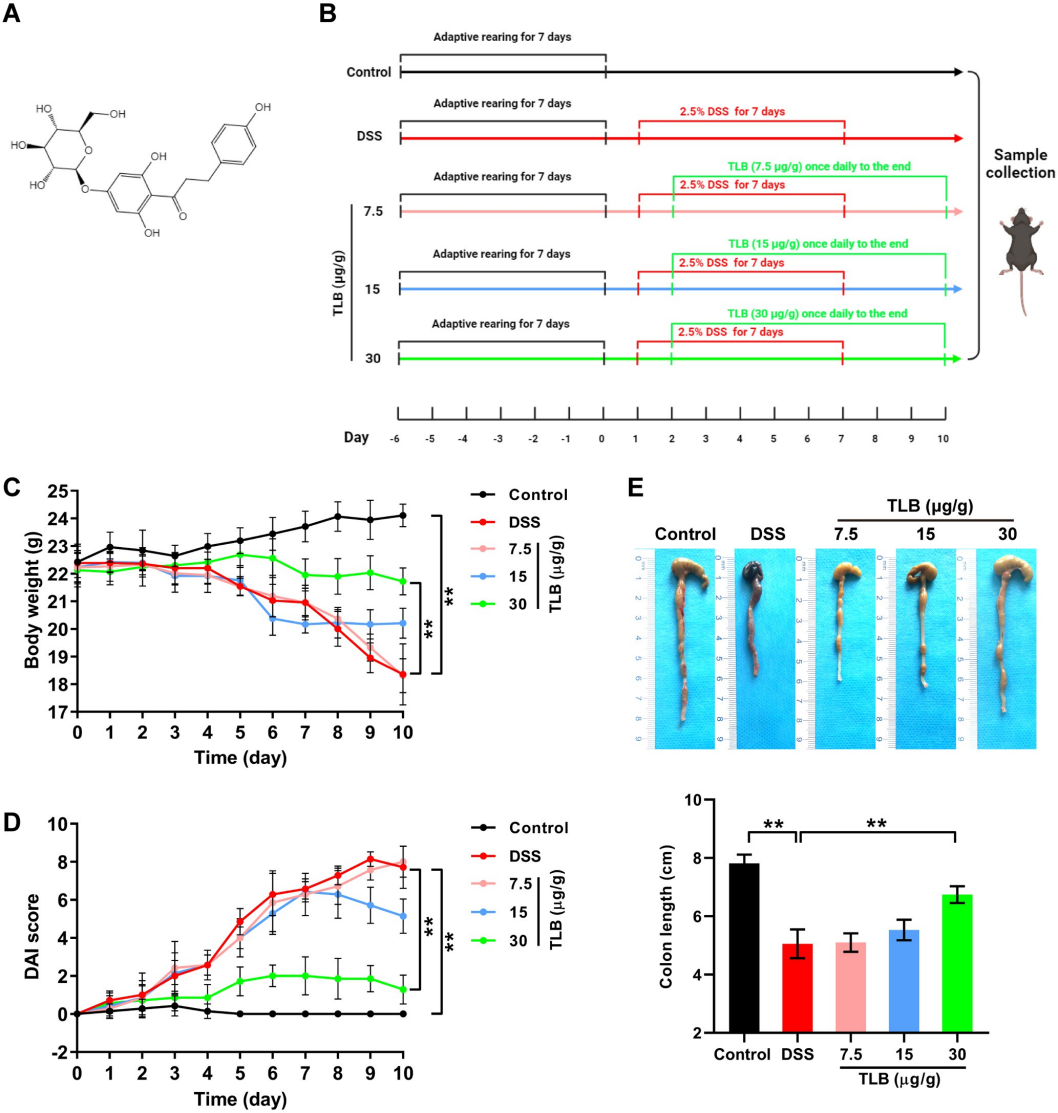
Effects of trilobatin (TLB)on the pathological symptoms of mice with dextran sodium sulfate (DSS)-induced ulcerative colitis (UC). **(A)** Structural formula of TLB. **(B)** Schematic representation of the experimental design. **(C)** Body weight (g) measured daily. **(D)** Disease activity index (DAI) score. **(E)** Representative images of colon and colon length. Data are expressed as mean ± standard deviation (SD). ** P < 0.05 vs. control (n = 7).

In recent years, drugs based on natural products, which have few side effects but sound therapeutic effects, have been widely used to treat various diseases. Trilobatin (TLB; C_21_H_24_O_10_; Fig 1A), commonly known as a natural sweetener, is a dihydrochalcone derived from the leaves of *Lithocarpus polystachyus* Rehd [18]. Numerous studies have revealed the therapeutic effects of TLB in multiple disorders such as diabetes, acute lung injury, cerebral ischemia/reperfusion injury, and Alzheimer’s disease (AD). However, there are no reports on the role of TLB in the treatment of UC.

In this study, we evaluated the effects of TLB on mice with dextran sulfate sodium (DSS)-induced UC and investigated the underlying molecular mechanisms regulating potential signaling pathways and variations in gut microbial composition. Our goals were to verify the therapeutic effect of TLB on UC and provide experimental evidence to support the clinical application of TLB in the treatment of UC.

## Materials and methods

### Drugs and chemical reagents

Colitis-inducing grade DSS, used for the construction of a mouse model of UC was purchased from MP Biomedicals (Irvine, CA, USA). TLB was provided by MedChemExpress Co., Ltd. (Monmouth Junction, NJ, USA). ELISA kits for measuring the levels of TNF-α, IL-1β, and IL-6 were purchased from Beyotime Co., Ltd. (Shanghai, China). TRIzol, used for RNA extraction, was purchased from Thermo Fisher Co., Ltd. (Waltham, MA, USA). Both Hifair^®^ III 1^st^ Strand cDNA Synthesis SuperMix for qPCR and cDNA synthesis and Hieff^®^ qPCR SYBR Green Master Mix for qPCR analysis were purchased from Yeasen Biotech Co., Ltd. (Beijing, China). Column tissue and cell protein extraction kits for protein extraction were purchased from Epizyme Co., Ltd. (Shanghai, China). Primary antibodies used in western blotting analysis included: anti-ZO-1, anti-occludin, anti-phosphorylated p65 (p-p65), anti-total p-65 (t-p65), anti-phosphorylated IKB-α (p-IKB-α), and anti-total IKB-α (t-IKB-α) purchased from Abcam Co., Ltd. (Cambridge, UK); anti-phosphorylated Akt (p-Akt) and anti-total Akt (t-Akt) purchased from Affinity Biosciences Co., Ltd. (Cincinnati, OH, USA); and anti-β-actin purchased from ProteinTech Co., Ltd. (Chicago, IL, USA). Secondary antibodies used for western blotting and horseradish peroxidase-conjugated Affinipure Goat Anti-Rabbit/Mouse IgG were purchased from ProteinTech Co., Ltd. (Chicago, IL, USA). The BCA protein assay kit used for assessing protein concentration and BeyoECL Moon (ECL kit) were purchased from Beyotime Co., Ltd. (Shanghai, China).

### Construction of ulcerative colitis model of mice

All animal procedures were performed in accordance with the Ethics Committee of the Changchun University of Chinese Medicine (Approval No. 2023161). All animal research staff members were trained in animal care and handling. Male C57BL/6 mice (6–7 weeks old, 20–22 g) were obtained from Shenyang Changsheng Company (Approval No. SCXK 2020–0001; Shenyang, China) was used to establish the UC model [19]. Prior to the treatment of DSS, the mice were randomly divided into five groups (n = 7) after a7-d adaptive rearing period at 18–23 °C under a 12-h light/dark photoperiod. The groups were as follows: the control group (without treatment of DSS and TLB), the DSS group (without treatment of TLB), and three TLB groups (treated with TLB at the dosage of 7.5, 15, and 30 μg/g, respectively). Besides the control group, the other four groups were continuously administered 2.5% (w/v) DSS in drinking water for 7 d. TLB was administered by gavage once daily from day 2 to the end of the experiment (Fig 1B; created with BioRender.com/; accessed on 10 September 2023). During the experiment, the body weight, stool properties, and hematochezia of mice were recorded daily.

### Analysis of UC disease activity analysis of ulcerative colitis

Disease activity index (DAI) scores combined with weight loss, stool characteristics, and hematochezia scores, were calculated to assess UC severity in mice. The scores for each factor were ranked as follows. Weight loss: 0, no change; 1, ≤ 5%; 2, > 5% and ≤ 10%; 3, > 10% and ≤ 15%; and 4, > 15%. Stool characteristics: 0 = normal; 1 = soft stool; 2 = moderate diarrhea; and 3 = diarrhea. Hematochezia: 0 = no rectal bleeding; 1 = slight rectal bleeding; 2 = bloodstain visible on stool; and 3 = visible rectal bleeding. The DAI score was the sum of the weight loss, stool characteristic, and hematochezia scores [20].

### Sample collection

At the end of the experiment, all mice were anesthetized with isoflurane vapor to collect blood samples for the detection of inflammatory mediators in the serum by eyeball enucleation. All mice were then euthanized by isoflurane vapor followed by cervical dislocation. Colon tissues were collected for measurement of colon length, histopathological examination, and extractions of total RNA and proteins for gene expression analysis. During stool sample collection for gut microbiota analysis, the mice were housed individually. All efforts were made to minimize animal suffering, with early humane endpoints criteria specified by the Institutional Animal Ethics Committee to determine when animals should be euthanized, including when one or more of the following were true: loss of > 25% of body weight, labored breathing, lack of response to stimuli, and inability to move. Animal health and behavior were monitored every 12 h. Once the animals reached the endpoint, they were euthanized immediately. None of the mice died before the humane endpoint criteria for euthanasia were met.

### Histopathological examination

Hematoxylin-eosin (HE) staining was performed for the histopathological examination of the colon tissue. The colon tissues were first fixed in 10% neutral formalin solution, and then paraffin-embedded, sectioned (5-μm thickness), stained with HE, observed for pathological variations, and photographed with an inverted microscope (magnification, × 200).

### ELISA analysis

ELISA was performed to detect the levels of three serum inflammatory factors (TNF-α, IL-1β, and IL-6) in mice. Following the ELISA kit manufacturer’s instructions, the standard samples and serum samples were added to the wells. The biotinylated antibody, horseradish peroxidase-streptavidin, TMB chromogen solution, and stop solution were sequentially added to wells containing the samples. The absorbance values were measured at 450 nm and used to generate a standard curve, which was used to calculate the concentrations of inflammatory factors.

### Quantitative real-time PCR

Quantitative real-time PCR (qRT-PCR) was performed to detect mRNA expression levels. Total RNA was extracted from colon tissue using TRIzol reagent. According to the manufacturer’s instructions, mRNA was reverse-transcribed into cDNA for qRT-PCR using a SYBR Green kit. Each sample was run in triplicate, with β-actin used as the internal reference. The relative mRNA expression levels of the target genes (encoding TNF-α, IL-1β, IL-6, ZO-1, and occludin, respectively) was calculated using the 2^–ΔΔCt^ method [21]. The primers and sequences used for qRT-PCR are listed in Table 1.

**Table 1:** Primers and their sequences used for qRT-PCR.

Primer	Sequence (5’→3’)
TNF-α	F: GGGCCATAGAACTGATGAGAGGG R: TCCAGAACTCCAGGCGGT
IL-1β	F: GTAGTGCAGTTGTCTAATGGGAACG R: GCTACCTGTGTCTTTCCCGTGGA
IL-6	F: TTGTATGAACAACGATGATGCACT R: TGTTCTTCATGTACTCCAGGTAGC
ZO-1	F: CACTACAGTATGACCATCCTCA R: TTCTCTGTTCACACAGATAAGC
Occludin	F: TTATACTCCTGCAGACCTGCATCAA R: GACGGACCCTGACCACTATGAAACA
β-actin	F: TACCACCAGACAGCACTGTGTT R: GAGGCTCTTTTCCAGCCTTCCTT

### Western blotting analysis

Western blotting analysis was used to detect the expression levels of target proteins (i.e., ZO-1, occludin, t-p65, p-p65, t-Akt, p-Akt, p-IKB-α, t-IKB-α, and β-actin). Total proteins from the colon tissues were extracted using column tissue or cell protein extraction kits. The protein concentration was measured using a BCA kit. Protein samples were separated by SDS-PAGE and transferred from the gel a polyvinylidene difluoride membrane. After blocking, membranes were sequentially incubated with the corresponding primary and secondary antibodies. The proteins were detected using an ECL kit. The grayscale value of protein bands was calculated using ImageJ (version 1.53e), with β-actin used as the internal reference. The samples loaded in each lane were derived from a pool of equal protein samples from all the mice in each group. Data from three independent experiments were analyzed.

### Gut microbiota analysis

16S rRNA gene sequencing and data analysis were performed by Novogene Technology Co., Ltd. (Beijing, China). Bacterial genomic DNA was extracted from fecal samples of three groups of mice, i.e., the control, DSS, and one TLB group (with a TLB dosage of 30 μg/g), using a DNA isolation kit (TianGen, Shanghai, China). DNA quality was verified using agarose gel electrophoresis. Primers of the 16S rRNA gene V3-V4 region were used for DNA amplification. Amplification products were separated using a gel extraction kit for subsequent sequencing. Library was constructed using NEB Next^®^ Ultra^™^ II FS DNA PCR-free Library Prep Kit (New England Biolabs, Ipswich, MA, USA) and then sequenced on an Illumina NovaSeq 6000 (San Diego, CA, USA) platform. Raw data were processed by quality control, merging, filtering, and chimera removal to obtain effective tags, using FLASH (version 1.2.11) and fastp (version 0.23.1). Amplicon sequence variants (ASV) were obtained after denoising using the DADA2 module of QIIME2 (V 202006). ASVs were first annotated to taxonomic ranks, and then used to perform the α diversity, β diversity, linear discriminant analysis (LDA) effect size (LEfSe), and phylogenetic investigations of communities by reconstruction of unobserved states (PICRUSt) to evaluate the microbial abundance, composition, and diversity. Function prediction was performed using the Kyoto Encyclopedia of Genes and Genomes (KEGG) database with statistical processing performed using Python, R, and Perl.

### Statistical analysis

All quantified data were presented as the mean ± standard deviation (SD). The data were assessed for normality using the Shapiro–Wilk test and analyzed by one-way ANOVA, followed by the *post-hoc* Sidak test using the statistical package GraphPad Prism (version 8.0.1). The cut-off for statistical significance was set at *P* < 0.05.

## Results

### Effects of TLB on the pathological symptoms of mice with DSS-induced UC

A significant decrease in body weight was observed in UC mice compared with that of the control group (Fig 1C), while the body weight of TLB group (30 μg/g) was significantly higher than that of the DSS group. To further assess the overall severity of UC in mice, the DAI score was calculated. The highest DAI score was observed in the DSS group (Fig 1D). Colon length results showed a similar pattern to that of body weight in the different groups of mice (Fig 1E). The colon length of UC mice was significantly shorter than that of the control group, and TLB (30 μg/g) treatment significantly reversed the reduced colon length of DSS-induced UC mice. These data demonstrated that TLB alleviated the pathological symptoms of DSS-induced UC in mice.

### Effects of TLB on colonic inflammation and mucosal barrier damage in mice with DSS-induced UC

**Fig 2:**
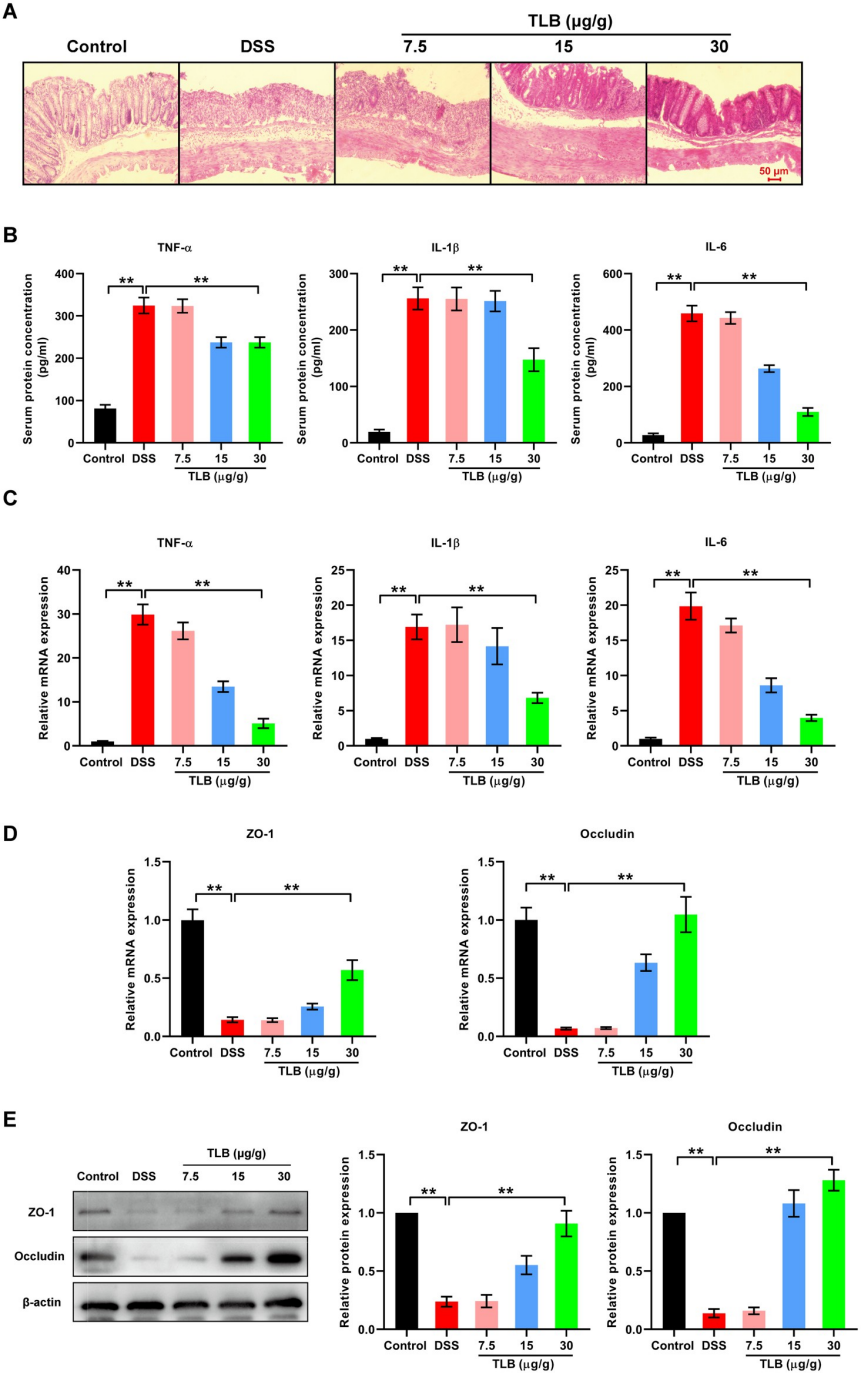
Effects of TLB on colonic inflammation and mucosal barrier damage in mice DSS-induced UC. **(A)** Representative images of hematoxylin-eosin (HE)-stained colon tissue sections (scale bar = 50 μm; magnification, × 200). **(B)** Expression levels of three inflammatory cytokines (TNF-α, IL-1β, and IL-6) in the sera of mice measured by ELISA. **(C)** Expression levels of genes encoding three inflammatory cytokines (TNF-α, IL-1β, and IL-6) in colon tissue measured by quantitative real-time PCR (qRT-PCR. **(D)** Expression of mRNA levels of genes encoding tight junction proteins (ZO-1 and occludin) in colon tissue measured by qRT-PCR. **(E)** Protein levels of tight junction proteins (ZO-1 and occludin) in colon tissue measured by western blotting analysis. Data are expressed as mean ± SD. ** P < 0.05 vs. control (n = 7).

To evaluate the effects of TLB on colonic inflammation and mucosal barrier damage in DSS-induced UC mice, indicators of inflammation and the mucosal barrier were assessed based on HE staining (Fig 2A). Compared to the control group, the DSS group showed the disappearance of the normal structure in the colon tissue (i.e., intact mucosa and crypts), which was infiltrated by large numbers of inflammatory cells. With increasing TLB concentrations, colonic inflammation and damage gradually decreased. With 30 μg/g TLB, the normal mucosal structure and crypt morphology were usually observable, besides small numbers of inflammatory cells. The secretion and expression of inflammatory cytokines (TNF-α, IL-1β, and IL-6) in serum and colon tissues were further detected. The ELISA results showed that levels of these inflammatory cytokines in the serum of DSS group were significantly up-regulated compared with the control group (Fig 2B), while the TLB (30 μg/g) treatment significantly inhibited the upregulation of inflammatory cytokines. Similarly, the qRT-PCR results showed that the expression levels of TNF-α, IL-1β, and IL-6 were significantly increased in the colon tissue of mice from the DSS group compared with the control group, whereas the elevated expressions of these inflammatory cytokines were significantly reversed by TLB (30 μg/g) treatment (Fig 2C). These results demonstrated that TLB could inhibit inflammation in mice with DSS-induced UC. The mRNA expression levels of tight junction proteins ZO-1 and occludin, which are related to colonic mucosal barrier damage, were also measured using qRT-PCR. The results showed that, compared with the control group, the mRNA levels of tight junction proteins were significantly decreased in the DSS group, while treatment with TLB (30 μg/g) significantly reversed the inhibited expression of these genes by DSS (Fig 2D). These protein expression patterns were consistent with qRT-PCR results (Fig 2E). These results suggested that TLB treatment protected against colonic mucosal barrier damage in mice with DSS-induced UC. Based on the effectiveness of the TLB (30 μg/g) treatment, this group of mice was selected in the subsequent experiments. Overall, TLB exhibited significant therapeutic effects against DSS-induced UC in mice.

### NF-κB pathway was inhibited by TLB in mice with DSS-induced UC

**Fig 3:**
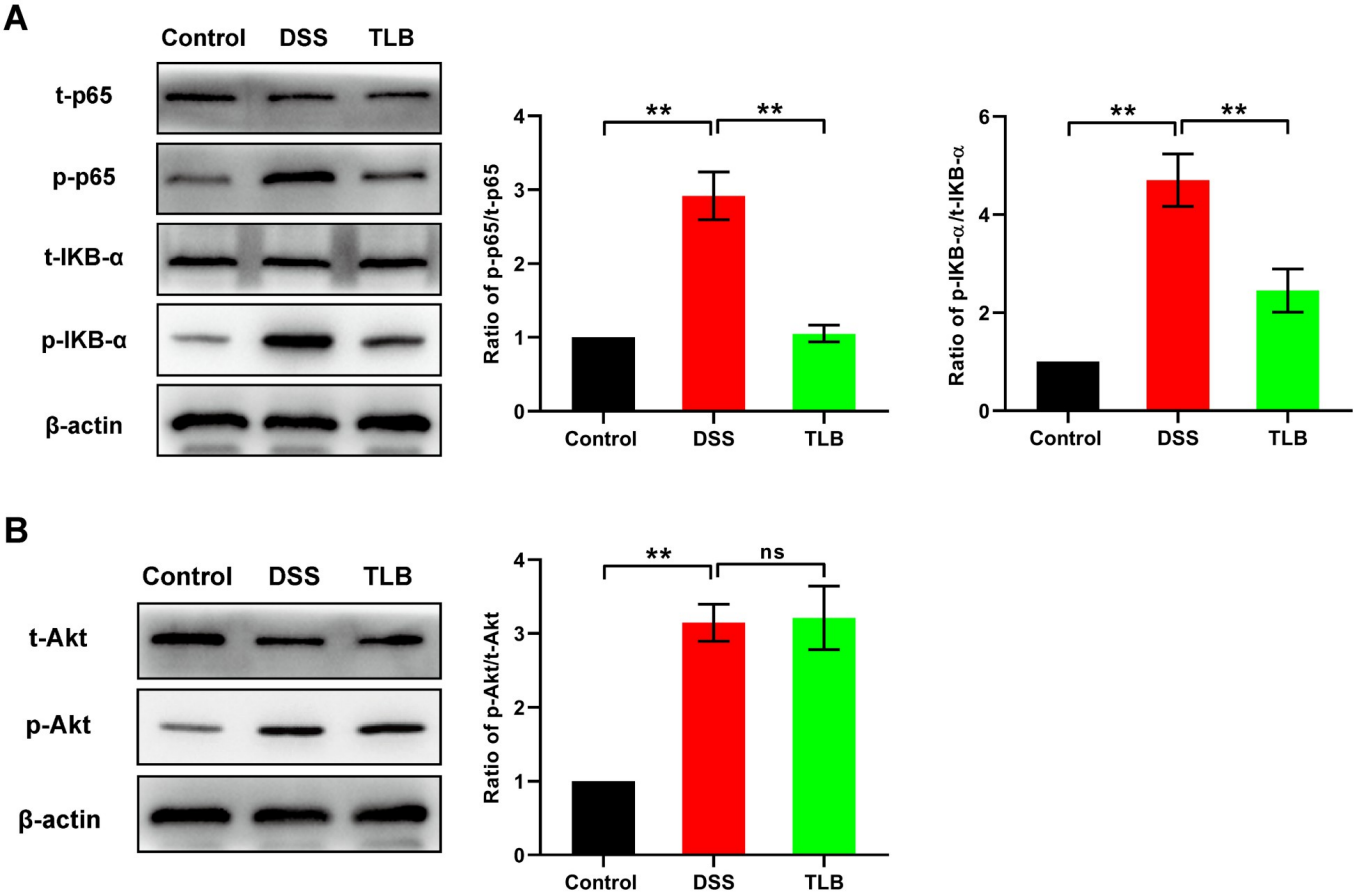
NF-κB pathway was inhibited by TLB in mice with DSS-induced UC. **(A)** Levels of the NF-κB pathway-associated proteins (p-p65, t-p65, p-IKB-α, and t-IKB-α) detected by western blotting analysis. **(B)** Levels of the PI3K/Akt pathway-associated proteins (p-Akt and t-Akt) detected by western blotting analysis. Data are expressed as mean ± SD. ** P < 0.05 vs. control. “ns” indicates no significant difference.

To further investigate the downstream variations caused by TLB-mediated therapeutic effects on DSS-induced UC mice, the activity of UC-associated signaling pathways, i.e., the NF-κB and PI3K/Akt pathways, were evaluated by the expression levels of the marker proteins, due to their promotion of the initiation and progression of UC. The results showed that the ratios of p-p65/t-p65 and p-IKB-α/t-IKB-α were increased in the DSS group compared with the control group, and these ratios were significantly decreased in the TLB group, indicating that TLB treatment suppressed the NF-κB pathway in DSS-induced UC mice (Fig 3A). No significant difference was detected in the p-Akt/t-Akt ratio in the PI3K/Akt pathway between the TLB and DSS groups (Fig 3B). These data suggested that TLB showed therapeutic effects on DSS-induced UC mice via the NF-κB pathway, instead of the PI3K/Akt pathway.

### Composition of gut microbiota modulated by TLB in mice with DSS-induced UC

**Fig 4:**
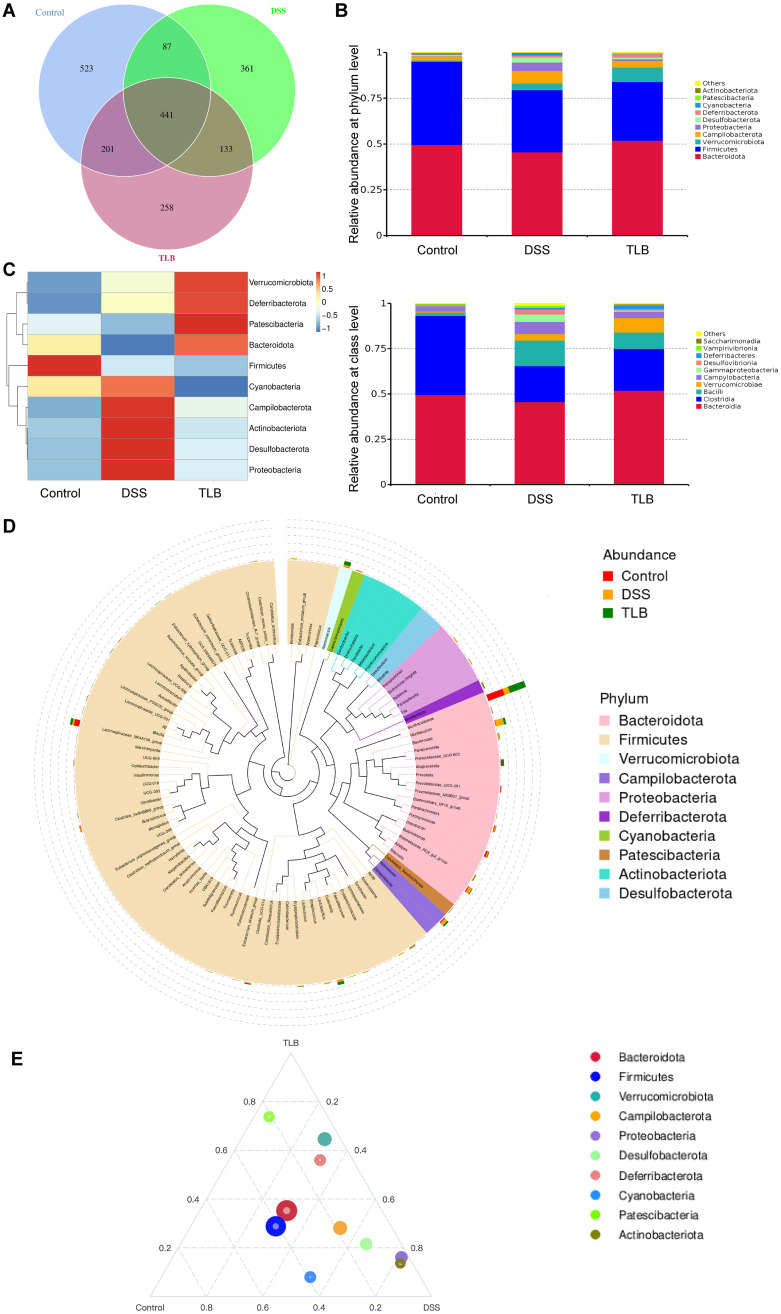
Composition of gut microbiota modulated by TLB in mice with DSS-induced UC. **(A)** Venn diagram showing the bacterial taxa in three groups of mice. **(B)** Average relative abundance of the top 10 taxa at the phylum or class levels. **(C)** Heatmap of microbial relative abundance and taxa clustering. **(D)** Phylogenetic tree of gut microbiota at the genus level. **(E)** Ternary plot of the top 10 microbial taxa.

Considering that the gut microbiota could significantly contribute to the regulation of UC development, 16S rRNA sequencing was performed to evaluate the effect of TLB on the gut microbiota composition in UC mice. A Venn graph was generated to show a total of 441 common microbial taxa shared among the three groups of mice, that is, the control, DSS, and TLB groups, and a total of 523, 361, and 258 unique taxa in the three groups, respectively (Fig 4A). The gut microbiota distribution was further investigated among these groups at the phylum and class levels (Fig 4B–4E). The results revealed that, compared to the control group, 5 of the top 10 bacterial phyla showing increased relative abundance included Campilobacterota, Proteobacteria, Desulfobacterota, Cyanobacteria, and Actinobacteriota in the DSS group; TLB treatment decreased the relative abundances of these phyla, whereas the relative abundances of Bacteroidota and Patescibacteria were decreased in the DSS group and increased by treatment with TLB. Five bacterial classes (i.e., Bacilli, Campylobacteria, Gammaproteobacteria, Desulfobacterota, and Vampirivibrionia) were found to have higher relative abundances in the DSS group than in the control group, but lower than in the TLB group, whereas both classes of Clostridia and Saccharimonadia showed lower relative abundances in the DSS group than in the control group, but higher than in the TLB groups.

### Relative abundance and diversity of gut microbiota modulated by TLB in mice with DSS-induced UC

**Fig 5:**
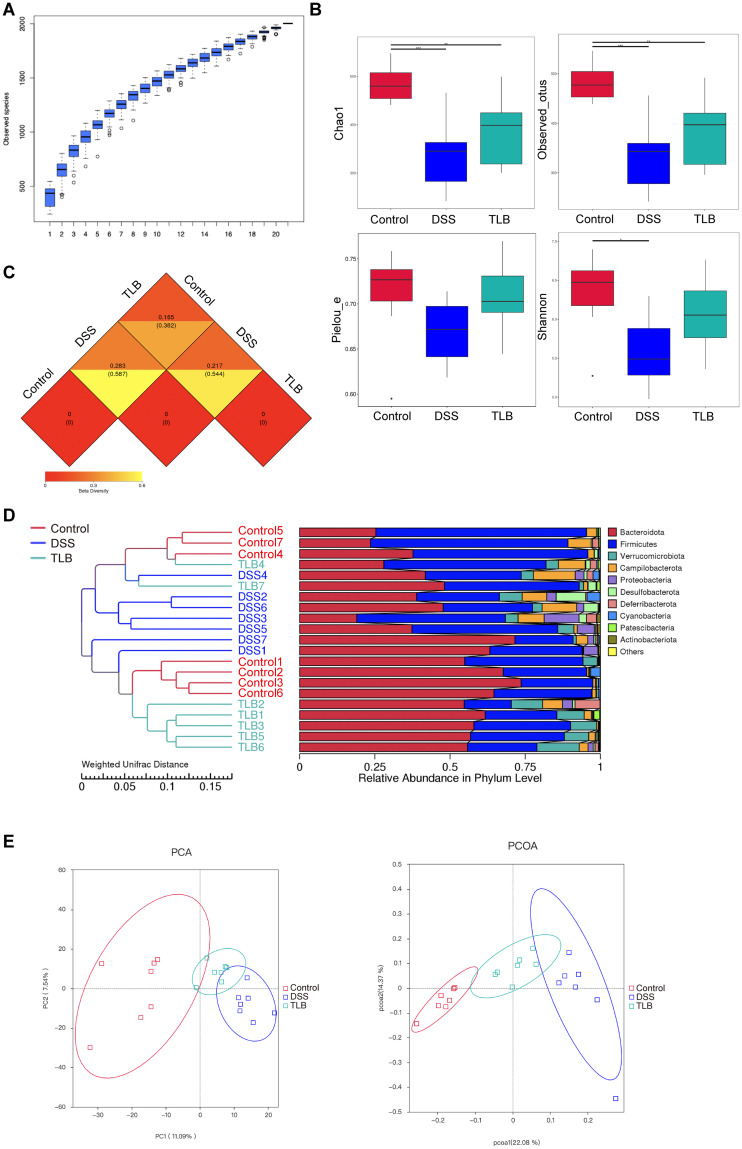
Relative abundance and diversity of gut microbiota modulated by TLB in mice with DSS-induced UC. **(A)** Species accumulation boxplot. **(B)** α diversity analysis based on the Chao1, observed_OTUs, Shannon, and pielou_e indices. **(C)** Distance matrix heatmap. **(D)** Hierarchical cluster tree based on the relative abundance of the top 10 bacterial phyla. **(E)** Bray–Curtis-based principal component analysis (PCA) and principal coordinate analysis (PCoA) plots based on unweighted UniFrac.

To further explore the microbial community characteristics of the three groups of mice, the relative abundance and diversity of the gut microbiota were evaluated. Species accumulation boxplots were generated to determine whether the sample size was sufficient for evaluating species richness. A trivial increase in the ASV values indicated that it was appropriate to perform subsequent analyses (Fig 5A). Compared to the control group, the values of Chao1, observed_OTUs, and Shannon indices were significantly decreased in the DSS group, while the value of the pielou_e index also declined in the DSS group, although this change was not statistically significant. The TLB administration increased these α diversity indices, suggesting that TLB affected the community richness of the gut microbiota in DSS-induced UC mice (Fig 5B). In our evaluation of β diversity in the gut microbiota, a heatmap was generated based on the distance matrix derived from the differences in species complexity of each pair of two groups of samples. The UniFrac distance factors between the control and TLB groups (0.615/0.382 for weighted/unweighted UniFrac, respectively) were lower than those of the other two pairwise comparisons (0.217/0.544 and 0.283/0.587, respectively) (Fig 5C). Hierarchical cluster trees indicated that DSS induction altered the microbiota structure, whereas TLB treatment partially recovered the microbial composition to the level of the control group (Fig 5D). Principal component analysis (PCA) and Principal coordinate analysis (PCoA) were performed to determine separation between the control and DSS groups, which indicated a difference in the main bacterial communities between the two groups. TLB group displayed close relatedness to the control group, showing that TLB treatment played a role in restoring bacterial taxa towards the normal state (Fig 5E). Both α and β diversity analyses suggested that TLB exerted its therapeutic effect by modulating the abundance and diversity of gut microbiota in DSS-induced UC mice.

### Differential bacterial taxa of gut microbiota modulated by TLB in mice with DSS-induced UC

**Fig 6:**
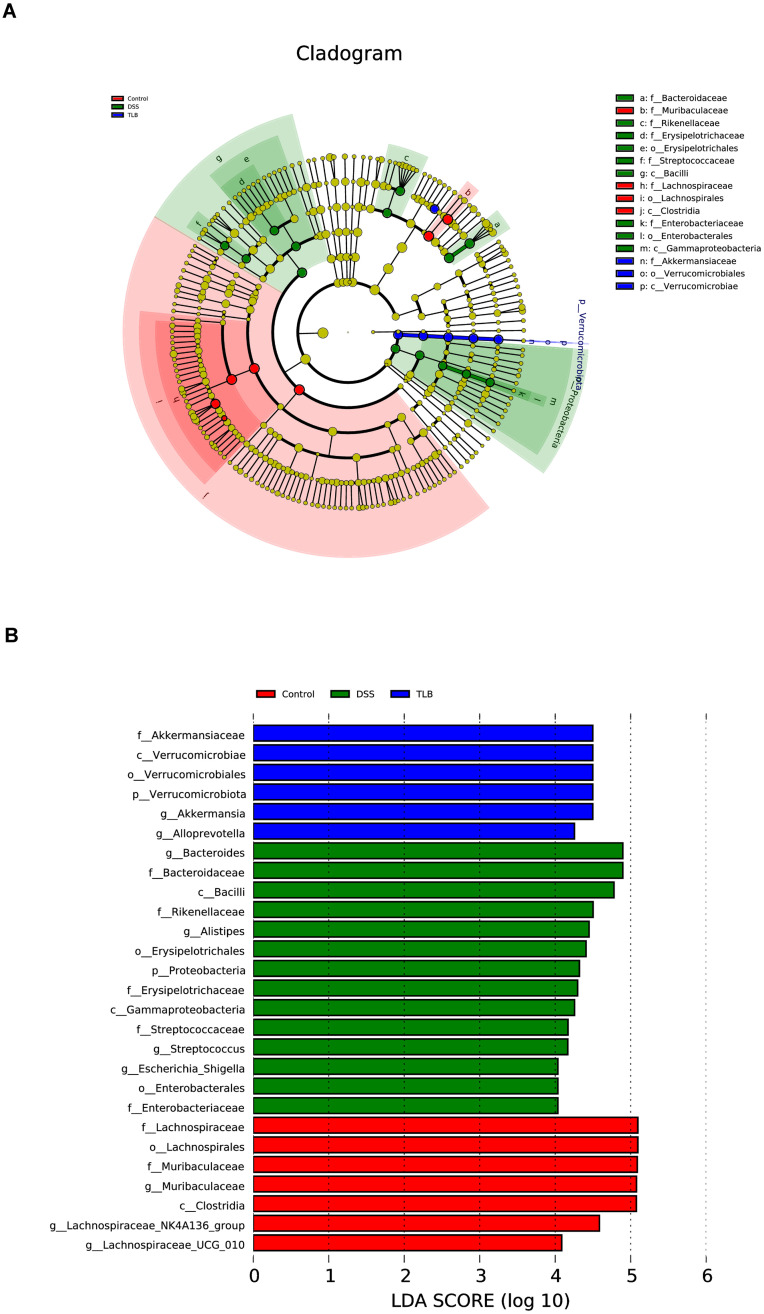
Specific differential bacterial taxa of gut microbiota modulated by TLB in mice with DSS-induced UC. **(A)** Taxonomic cladogram. **(B)** Linear discriminant analysis effect size (LEfSe) analysis of gut microbiota among the three groups of mice with linear discriminant analysis (LDA) score > 4.

To investigate the specific community variations in the gut microbiota among the three groups of mice, LEfSe with LDA scores and cladograms were generated. The results showed that there were 7, 19, and 6 differential bacterial taxa in the control, DSS, and TLB groups, respectively (Fig 6A and 6B). The relative abundances of g_Bacteroides, f_Bacteroidaceae, and c_Bacilli were high in the DSS group, while both f_Akkermansiaceae and c_Verrucomicrobiae were increased in the TLB group. These results showed that TLB treatment decreased the number of specific differential bacteria in DSS-induced UC mice.

### Functional prediction of gut microbiota modulation by TLB in mice with DSS-induced UC

**Fig 7:**
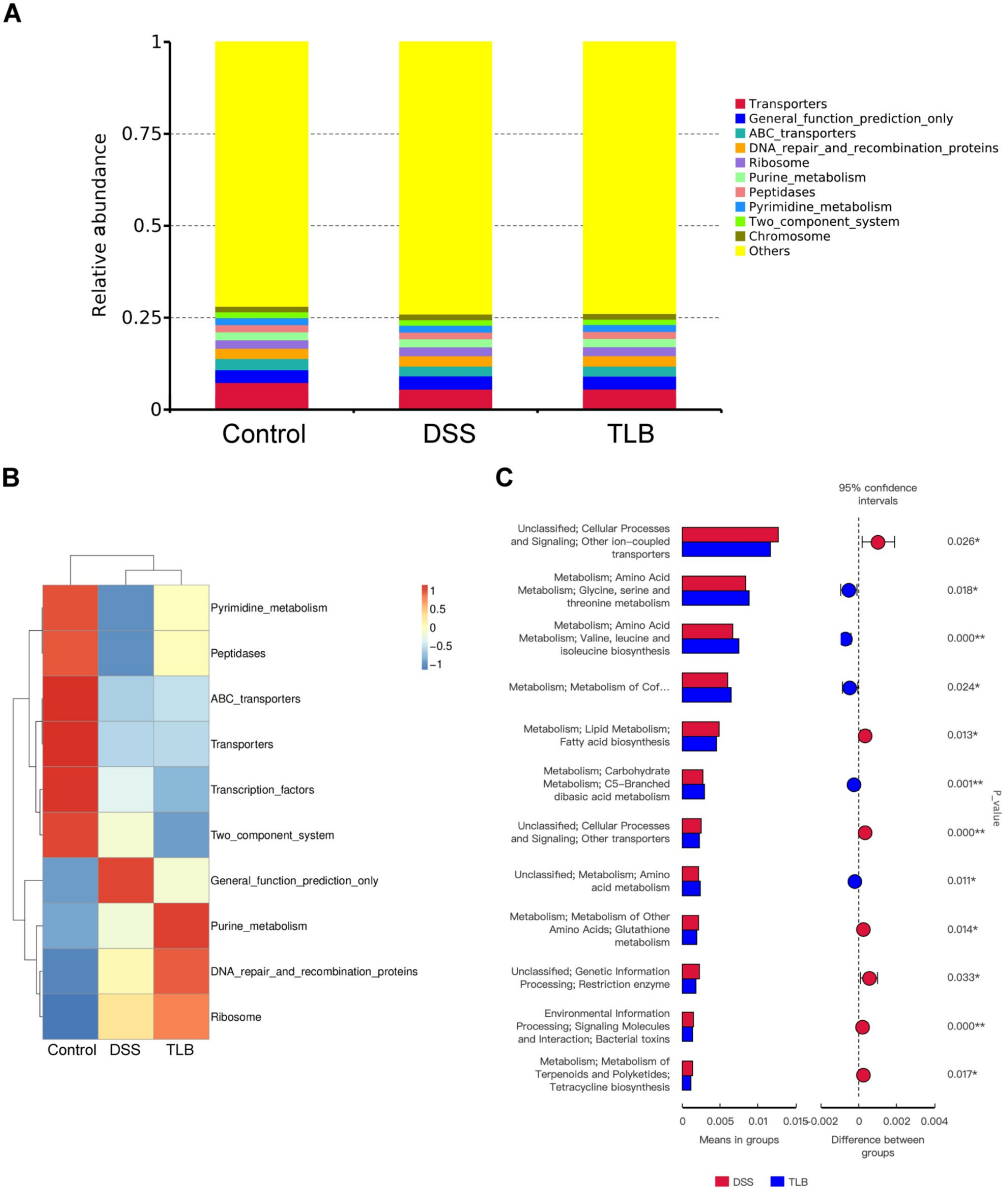
Functional prediction of gut microbiota modulation by TLB in mice with DSS-induced UC. **(A)** Relative abundance of function prediction annotation represented in the column. **(B)** Relative abundance of function prediction annotation represented in cluster heatmap. **(C)** Functional differences between groups based on *t*-test (*P* < 0.05).

To analyze the gut microbiota-associated functional alterations caused by TLB in DSS-induced UC mice, metagenomic prediction was performed using PICRUSt based on marker genes. The relative abundance and cluster heatmap results at KEGG level 3 revealed that the bacterial taxa in the DSS group were more associated with the “Chromosome” term and less associated with the terms “Transporter,” “Peptidases,” and “Pyrimidine_metabolism” than the other two groups of mice, while TLB treatment could reverse these variation patterns (Fig 7A and 7B). The functional diversity between DSS and TLB groups was validated by a t-test, showing that the variations were mainly detected in the “Metabolism” term (Fig 7C).

## Discussion

### Functions of natural flavonoids in UC

UC is a refractory digestive system disorder with no effective clinical treatment available at present. Recurrent symptoms and lifelong therapy for UC can lead to severe physical and economic stresses on the patients. One of the aims of UC therapy is to effectively control progression and ameliorate the symptoms of the disease. An increasing number of natural products reportedly have therapeutic effects on UC, providing new insights into UC treatment. Compared to traditional drugs, the administration of natural products has shown low toxicity and fewer side effects [22]. These extracts contain flavonoids, phenols, and alkaloids [23]. To date, the research on flavonoids for the treatment of UC has continuously identified new natural products with therapeutic effects against various diseases. For example, luteolin, a natural flavonoid, was found to alleviate spontaneous colitis [24], exerting anti-inflammatory effects by inhibiting iNOS and COX-2 levels in an azoxymethane-induced mouse model of spontaneous colitis [25]. Baicalin, another type of flavonoid derived from *Scutellaria baicalensis* Georgi, is capable of decreasing the severity of DSS-induced UC in mice by suppressing the levels of IL-33 and subsequently activating the NF-κB pathway [26]. Furthermore, a study on the role of baicalin in UC showed that it could alleviate inflammation by enhancing the proliferation of CD4^+^ and CD29^+^ T helper cells and inhibiting both the STAT and NF-κB pathways [27]. Moreover, myricetin, a natural flavonoid widely found in various plants, exerts protective effects against DSS-induced UC in mice by significantly ameliorating UC-related inflammatory and oxidative reactions [28]. The results of our study show for the first time that TLB, which is also a type of natural flavonoid, could ameliorate the pathological symptoms, inflammation, and colonic mucosal barrier damage in mice with DSS-induced UC. These findings indicate that TLB may be a valuable candidate for the clinical treatment of UC. Further studies are needed to verify the therapeutic application of TLB in the treatment of UC.

### Functions of TLB in various diseases

TLB, a natural sweetener, has constantly been found to play important roles in a wide range of diseases. Among the biological activities of TLB, its protective effects against tissue and cell injury, which are exerted via its anti-inflammatory and anti-oxidant properties have attracted our attention. Previous studies have shown that TLB protected against injury in a hydrogen peroxide-induced oxidative stress model of neuronal PC12 cells by regulating the AMPK/Nrf2/Sirt3 signaling pathway [18]. TLB was revealed to play a neuroprotective role in a rat model cerebral ischemia/reperfusion injury by inhibiting inflammatory and oxidative responses via the TLR4/NF-κB and Nrf2/Keap-1 pathways [29], and promoting the proliferation of cerebral microvascular endothelial cells and angiogenesis via the SIRT7/VEGFA pathway [30]. In studies of a mouse model of AD, TLB showed anti-inflammatory effects by targeting HMGB1 and regulating the Sirt3/SOD2 pathway [31], and reducing the expression levels of TNF-α, IL-1β, and IL-6 via TLR4/MYD88/NF-κB pathway [32]. In isoflurane-induced injury in mouse hippocampal neuronal HT22 cells, TLB exerted protective effects by activating the Nrf2/ARE signaling pathway [33]. In lipopolysaccharide-induced acute lung injury in mice, TLB treatment effectively inhibited the oxidative stress and inflammatory damage via the AMPK/GSK-3β/Nrf2 and NF-κB pathways [34]. These findings promoted us to evaluate the role of TLB in UC, an inflammatory disease that frequently leads to extensive colonic damage. Our study adopted the classical animal model, mice with DSS-induced UC, for *in vivo* evaluation of the function of TLB. These results validated our speculation that TLB effectively alleviated DSS-induced UC in mice, indicating the extensive anti-inflammatory and anti-injury effects of TLB, and providing guidance for the treatment of related medical disorders. A recent study revealed the therapeutic effects of TLB on a diabetic mouse model induced by high-fat diet combined with streptozotocin [35]. Furthermore, a study on the role of TLB in human hepatoblastoma HepG2 and Huh-7 cells showed that TLB promoted the proliferation of cancer cells [36]. These data indicate that further studies are required to explore the biological activities of TLB.

### Effects of TLB on UC by regulating associated signaling pathways and gut microbiota

We further investigated the signaling pathways involved in the anti-UC effects of TLB. The results showed that TLB reversed the activation of the NF-κB pathway in mice with DSS-induced UC. These results are consistent with those of previous reports, revealing the role of TLB in inflammatory injury [29,32,34]. The NF-κB pathway is one of the main signaling pathways controlling the molecular network leading to the production of UC-associated inflammatory factors, suggesting the potential of NF-κB pathway as the therapeutic target [10]. Indeed, studies on UC treatment have revealed the therapeutic effects of several agents, such as baicalin, which suppresses the NF-κB signaling pathway (Zhang et al., 2017; Yu et al., 2014). Combined with the TLB-mediated inhibitory effects on the NF-κB pathway, we reasonably speculated that TLB played important biological roles as an inhibitor of the NF-κB signaling pathway. Notably, TLB exerts various effects on the PI3K/Akt pathway. In particular, previous studies have shown that activation of the PI3K/Akt pathway is associated with the expression of inflammatory factors in UC [37], while the phosphorylation of Akt also activates the NF-κB signaling pathway in UC [10]. However, our results revealed no significant effect of TLB on the activation of the PI3K/Akt pathway in mice with DSS-induced UC. Therefore, further investigations are needed to explore the explicit mechanisms underlying the signaling pathways involved in the TLB-mediated therapeutic effects on UC.

In the past decade, numerous clinical and experimental studies have demonstrated that gut microbiota play an important role in the regulation of UC development. For example, lower relative abundances of Lactobacilli and Faecalibacterium and higher relative abundances of Bacteroides and Veillonella were detected in a cohort of 48 patients with UC [38], while the gut microbiota was less diverse in patients with UC who either had active symptoms or were in remission compared to those in healthy individuals [39]. Furthermore, variation was detected in β diversity, but not in α diversity, between participants with active UC and those in short remission [40]. Therefore, the development of intestinal diseases, including inflammatory bowel disease (IBD) and tumors, is closely related to alterations in the gut microbiota. Several studies have reported that naturally active compounds show significant potential for the development of novel drugs against UC based on pharmacodynamic studies of the gut microbiota [41]. Furthermore, studies have shown that extracts of *Abelmoschus manihot* could alleviate UC in mice by modulating the gut microbiota composition by increasing the relative abundance of Firmicutes and decreasing the relative abundance of Bacteroidetes at the phylum level, whereas there were no significant differences in Proteobacteria [42]. Moreover, betaine treatment increased the relative abundance of Bacteroidota and Campylobacterota in UC mice [43]. In our study, TLB treatment reduced the relative abundance of Proteobacteria and Campylobacterota in mice with DSS-induced UC. Proteobacteria were previously considered an indicator of gut microbiota dysbiosis, which is closely related to metabolic disorders [44], and was regarded as a predisposing factor for the onset of inflammatory bowel diseases (IBDs) [45], whereas Campylobacterota is associated with various intestinal diseases, including IBDs, enteritis, and colorectal cancer [46]. These results are consistent with those of the present study, showing that TLB is capable of alleviating intestinal inflammation by inhibiting the growth of Campylobacterota and promoting the abundance of Bacteroidota. Indeed, TLB affects the gut microbiota of high-fat diet induced obese rats [47] by increasing the relative abundance of Bacteroidota, which is consistent with our results. It has been reported that Bacteroidota play beneficial roles by providing energy via metabolizing glycose, and its shortage is associated with several diseases [48]. Therefore, the therapeutic effects of TLB on UC and obesity are probably associated with improved growth of Bacteroidota. Previous studies showed that the analysis of functional activities revealed that the “Metabolism” term was involved in many signaling pathways with TLB treatment [47]. The metabolites of the gut microbiota are important bridges connecting the microbiota to the host. Studies have shown that TLB modulates the metabolism of short-chain fatty acids (SCFAs), including propionic and butyric acids, in obese mice [47]. SCFAs function as nutrients that can be absorbed and utilized by the host and play important biological roles in the development of UC [49]. Therefore, it is necessary to further investigate the effect of TLB on the interactions between gut microbiota and metabolites in UC mice. In the present study, TLB decreased the relative abundance of Actinobacteriota in mice with DSS-induced UC. Previous studies reported that Actinobacteriota was abundant in both patients with active and inactive UC, based on a machine learning approach [50]. Although they represent less than 10% of the gut microbiota, Actinobacteriota was involved in many biological functions [51]. Patients with IBD were reported to show a decrease in the relative abundance of Firmicutes [52], the main components of the gut microbiota. However, our results revealed that TLB did not decrease the relative abundance of Firmicutes in mice with DSS-induced UC. Overall, our findings uncovered the molecular mechanism underlying the effects of TLB on mice with DSS-induced UC, which occurs via the modulation of the gut microbiota and enriched our knowledge of the regulation of gut microbiota by TLB.

## Conclusion

**Fig 8:**
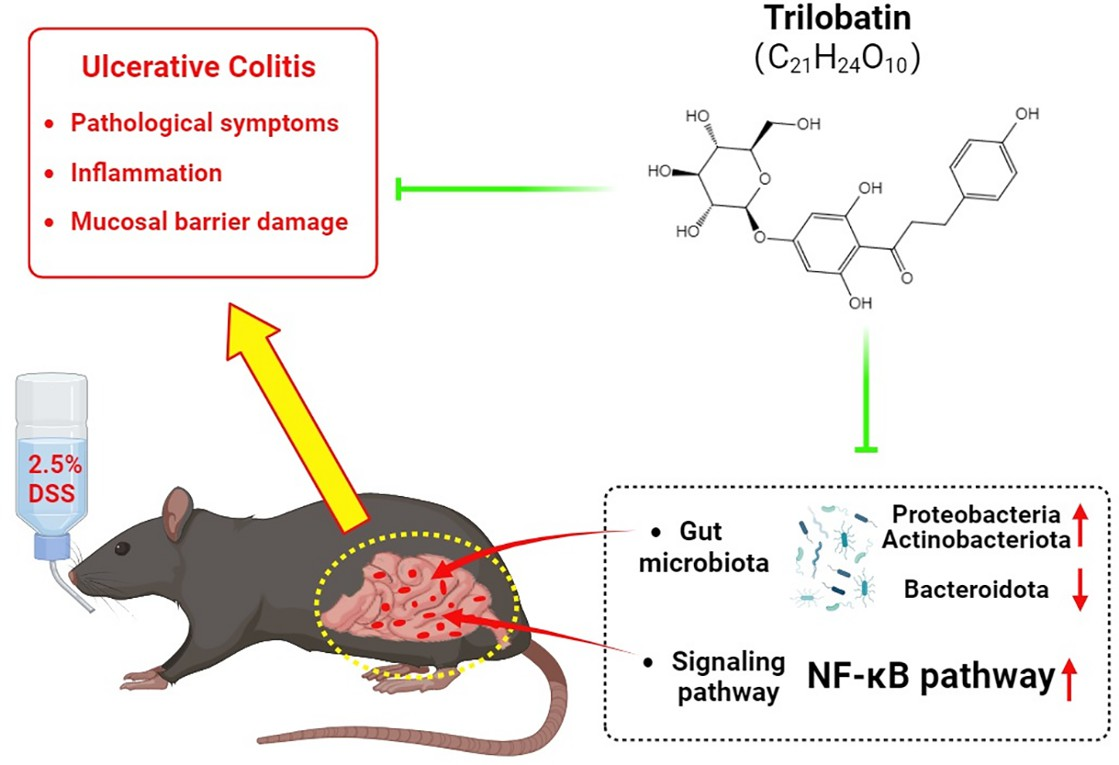
Schematic illustration of the main findings reported in this study. TLB alleviates the pathological symptoms, inflammation, and colonic mucosal barrier damage in mice with DSS-induced UC via the inhibition of the NF-κB pathway and alteration of gut microbiota composition (created with BioRender.com).

TLB could alleviate the pathological symptoms, inflammation, and colonic mucosal barrier damage in mice with DSS-induced UC by inhibiting the NF-κB pathway and altering the gut microbiota composition in mice. These findings (Fig 8) not only support TLB as a novel candidate agent for UC treatment, but also enhance our understanding of the role played by TLB and the underlying molecular mechanisms regulating its biological activities.

## Abbreviations

ASV: amplicon sequence variant
DAI: disease activity index
DSS: dextran sodium sulfate
HE: hematoxylin-eosin
IBD: inflammatory bowel disease
LDA: linear discriminant analysis
LEfSe: linear discriminant analysis effect size
p-Akt: phosphorylated Akt
PCA: principal component analysis
PCoA: principal coordinate analysis
PICRUSt: phylogenetic investigation of communities by reconstruction of unobserved states
p-IKB-α: phosphorylated IKB-α
p-p65: phosphorylated p65
qPCR: quantitative real-time polymerase chain reaction
SCFAs: short-chain fatty acids
SD: standard deviation
t-Akt: total Akt
t-IKB-α: total IKB-α
TLB: trilobatin
t-p65: total p-65
UC: ulcerative colitis
